# Review of the evidence on the influence of Wi-Fi 2.4 GHz radiation on oxidative stress and its possible relationship with Alzheimer’s disease

**DOI:** 10.3389/fneur.2025.1616435

**Published:** 2025-10-03

**Authors:** David Laván, Natalia Argüelles, Alexis Lluncor, Daniel Huaman, Juan Moyano, Jorge Ubillus, Milton Peña, Mónica Paredes, Iván Hernández, Alcides Guerra, Jhony De La Cruz-Vargas, Víctor Cruz

**Affiliations:** ^1^Instituto de Investigaciones en Ciencias Biomédicas, Facultad de Medicina Humana, Universidad Ricardo Palma, Lima, Peru; ^2^Department of Research, Development and Innovation, BioTechCell SAC, Lima, Peru; ^3^Faculty of Natural and Mathematical Sciences, National University Federico Villareal, Lima, Peru; ^4^Escuela de Medicina Humana, Universidad San Martín de Porres, Chiclayo, Lambayeque, Peru; ^5^Escuela de Ingeniería Electrónica, Facultad de Ingeniería, Universidad Ricardo Palma, Lima, Peru; ^6^Instituto de Investigación de Ciencias Biológicas “Antonio Raimondi”, Facultad de Ciencias Biológicas, Universidad Nacional Mayor de San Marcos, Lima, Peru; ^7^Escuela de Biología, Facultad de Ciencias Biológicas, Universidad Ricardo Palma, Lima, Peru; ^8^Facultad de Medicina Humana, Universidad Nacional de Ucayali, Ucayali, Peru

**Keywords:** Alzheimer’s disease, oxidative stress, electromagnetic radiation, Wi-Fi exposure y neurodegeneration, neuronal damage

## Abstract

To date, there is no scientific consensus on whether wireless communication systems, such as 2.4 and 5 GHz Wi-Fi, play a crucial role in the development of Alzheimer’s disease through oxidative stress. Although numerous studies have linked oxidative stress to exposure to electromagnetic radiation from wireless systems in various biological contexts, these studies have not established a direct connection to neurodegenerative diseases. Research on Alzheimer’s disease and oxidative stress is an active field in neuroscience and medicine, as oxidative stress involves an imbalance between the production of free radicals and the antioxidant system’s ability to neutralize them, leading to cellular and neuronal damage. It is essential to recognize that Alzheimer’s disease is multifactorial, and its development generally results from a complex interaction of genetic, environmental, and lifestyle factors. The relationship between wireless systems such as Wi-Fi and oxidative stress, as well as its possible link to Alzheimer’s disease, continues to be the subject of research and debate in the scientific community. Although some studies have explored this possible association, the results have been mixed and inconclusive. While research on the health effects of wireless systems remains relevant, it is prudent not to consider this association as an established fact until solid and consistent scientific evidence is available. The study we present focuses on indirectly analyzing the relationship between genes that respond to oxidative stress upon exposure to Wi-Fi 2.4 GHz electromagnetic waves and genes associated with the development of Alzheimer’s disease. Our results indicate that the modification of key genes involved in neurodegeneration, such as *GSK3B* and *APOE*, could be exacerbated by prolonged exposure to this radiation. It is essential for future research to explore this hypothesis to further clarify the potential risks associated with electromagnetic radiation and its impact on neuronal health and the progression of Alzheimer’s disease.

## Introduction

To assess the health impact of exposure to Wi-Fi radiation, it is essential to first understand the physical principles governing the propagation of radiofrequency waves, which are fundamental to telecommunications. These principles are based on Maxwell’s equations, developed by James Clerk Maxwell, which describe electromagnetic fields (EMF). This system of equations covers electromagnetic phenomena observable at a macroscopic scale in nature. Maxwell’s equations model the interaction between electric and magnetic fields, which propagate simultaneously at the speed of light. Although the frequency of the wave is directly related to the amount of energy it carries, this does not necessarily translate into greater bandwidth.

In particular, time-domain solutions of Maxwell’s equations in homogeneous and isotropic media are crucial for analyzing radiofrequency broadband phenomena in telecommunications. The solution to these equations employs advanced mathematical tools commonly used in modern physics. The Cartesian multipole expansion in the time domain enables the derivation of semi-analytical solutions for arbitrary, localized, and time-varying current distributions, thereby facilitating analysis in arbitrary planar geometries (1).

Broadband technology enables high-speed transmission of voice, video, and data over Information and Communications Technology (ICT) networks and applications. Prominent broadband technologies include Wi-Fi, as well as 4G and 5G cellular technologies. These technologies are deployed across various infrastructures, including community antennas, optical fiber, satellites, and fixed and mobile wireless systems. The deployment of these technologies has facilitated the global expansion of traditional and emerging telecommunications (2).

As telecommunications advances rapidly, broadband access technologies have emerged that offer comparable or superior performance to traditional network solutions in industrial or workplace environments (3). The IEEE 802.15.4a standard, designed for wireless personal area networks (WPAN) applications in industrial environments, has raised interest regarding its health implications due to its use in wireless data transmission at specific frequencies. This standard operates in 2.4 GHz, 868 MHz, and 915 MHz frequency bands, using signal modulation techniques for data transmission.

Although the exposures from Wi-Fi radiation are within the limits set by international regulations for exposure to electromagnetic fields, it is crucial to examine their potential impacts on public health. Guidelines from bodies such as the International Commission on Non-Ionizing Radiation Protection (62) and the International Electrical and Electronic Engineering Institute (63) have set exposure limits to protect human health from the potential adverse effects of electromagnetic fields based on acute heating. Non-thermal bioeffects are deemed to have no health impact. Exposure levels generated by devices using the IEEE 802.15.4a standard are typically well below these limits (4) thereby reducing the potential risk of adverse effects. Recent research suggests that exposure to 5G electromagnetic radiation (EMR) at 28 GHz may exert melanogenic effects by reducing melanin synthesis and attenuating the production of reactive oxygen species (ROS) in both murine and human skin cell models. In these studies, 5G exposure was found to decrease the activity of key enzymes involved in melanogenesis such as tyrosinase, TRP-1, and TRP-2 and to reduce ROS generation induced by stimuli like *α*-MSH or hydrogen peroxide, indicating a potential interference with oxidative mechanisms associated with skin pigmentation (5).

In contrast, studies conducted with electromagnetic radiation at 2.45 GHz a frequency commonly used in 4G and Wi-Fi technologies have reported cytotoxic and pro-oxidative effects, particularly in human SH-SY5Y neuronal cells and peripheral blood mononuclear cells (PBMCs). In these models, cell viability was significantly reduced after 24 to 48 h of exposure, and a marked increase in ROS levels was observed at all evaluated time points (6, 7).

In relation to Wi-Fi, the research group led by Aït-Aïssa exposed rats to Wi-Fi 2.45 GHz signals during gestation and early life stages. Subsequently, various indicators were analyzed in the rats’ sera and changes in gestational outcomes were assessed. Serum screening was performed to detect antibodies against 15 different antigens related to damage and/or pathological markers by enzyme-linked immunosorbent assay (ELISA). The results showed no alterations in the humoral response of the offspring, regardless of the type of biomarker or specific absorption rate (SAR) levels used. In addition, the study assessed gestational parameters following in utero exposure to Wi-Fi signals, including the mass of the mothers and offspring, the number of offspring per litter, and the genital tract of young offspring was examined for possible abnormalities by measuring the anogenital distance. The findings indicated the absence of significant adverse effects of Wi-Fi exposure on parturition and general condition of the animals (8).

Akar et al. (9) investigated the effects of 2.45 GHz electromagnetic fields on the rat cornea and found a significant increase in anterior corneal epithelium thickness in the exposed group. While the epithelial area percentage was also higher, no significant differences were found between tissue compartments. Further histological and stereological studies are recommended. Several studies have shown that exposure to radiofrequency electromagnetic fields (RF-EMF), particularly at frequencies used by mobile devices, can induce an increase in oxidative stress in biological tissues. For example, Avci et al. (10) demonstrated that 1.8 GHz radiation significantly increases malondialdehyde (MDA) levels and decreases glutathione (GSH) concentrations in rats, suggesting an imbalance in the cellular redox system. Similarly, Aydin and Akar (11) observed that exposure to 900 MHz affects oxidative stress parameters in lymphoid organs, polymorphonuclear leukocytes, and plasma, with evidence of a systemic inflammatory response mediated by reactive oxygen species.

In addition to oxidative stress, changes have been documented in the expression of genes involved in apoptosis and cellular damage response mechanisms triggered by electromagnetic fields. Eker et al. (12) reported significant alterations in the gene expression of caspase-3, Hsp27, p38MAPK, and epidermal growth factor (EGF) in the eyes of rats exposed to 1800 MHz, suggesting activation of intracellular pathways related to stress and cell death. On the other hand, Şekeroğlu et al. (13) identified cytotoxic and genotoxic effects associated with GSM 1800 MHz exposure in both immature and mature rats, through observed changes in cell morphology and DNA fragmentation. These findings reinforce the possibility that prolonged exposure to high-frequency RF-EMF may have adverse effects on cellular and genetic health, particularly during developmental stages or in highly proliferative tissues.

Bektas demonstrated that exposure to 2.4 GHz Wi-Fi signals and mobile phones during gestation influenced human placenta and cord blood. Increased levels of 8-OHdG, MDA, PCO, and TOS were observed in cord blood and placenta in the mobile phone-exposed group. However, the Wi-Fi signal-exposed group did not show alterations in the oxidative stress parameters studied. Furthermore, the tail intensity and DNA tail moment in the mobile phone-exposed groups were higher than those observed in the control groups and the Wi-Fi-exposed group In conclusion, the study’s results indicated that exposure to mobile phones during pregnancy may have a significant potential to cause oxidative stress and DNA damage in cord blood and the placenta. It was also found that the combination of Wi-Fi and mobile phone exposure might have a higher potential to cause synergistic harmful effects (14).

Another relevant study was conducted by Dasdag, who investigated the effects of prolonged exposure to 2.4 GHz radiofrequency (RF) radiation emitted by Wi-Fi equipment on testicular functions. The study concluded that prolonged exposure to 2.4 GHz radiation at a specific absorption rate (SAR) of 2,420 mW/kg for 1 g of tissue adversely affects several reproductive parametersin male rats. Based on these findings, Wi-Fi users are advised to limit their prolonged exposure to RF emissions from Wi-Fi devices (15).

The research group led by Jafari evaluated the impact of 2.4 GHz Wi-Fi radiation exposure on histopathological changes in the cardiovascular system in rats. The results indicated that heart weight and myocardial volume density were increased in the Wi-Fi-exposed group compared to the control group. Furthermore, Wi-Fi exposure led to an increase in malondialdehyde (MDA) levels, a decrease in total antioxidant capacity (TAC), and a reduction in reduced glutathione (GSH) levels. The study concluded that radiofrequency radiation (RFW) can induce structural modifications and oxidative stress in cardiac tissue, as well as cause myocardial hypertrophy and a reduction in myocyte number (16).

Complementing these findings, Kamali’s work provided additional evidence on oxidative stress induced by prolonged exposure to Wi-Fi radiation in rats. Kamali highlighted that increased exposure to electromagnetic radiation (EMR) in the everyday environment could have public health implications, with oxidative stress emerging as a relevant mechanism in the pathophysiology of various diseases. Until now, the impact of continuous exposure to radiofrequency radiation emitted by Wi-Fi hotspots on antioxidant redox systems in animal models had not been documented. The Kamali study exposed male Wistar rats to 2.45 GHz radiofrequency (RF) radiation from a commercial Wi-Fi device for 24 h a day for 10 weeks. Changes in the plasma antioxidant redox system were assessed by measuring total antioxidant capacity, thiobarbituric acid-reactive substances (TBARS) levels, reduced glutathione (GSH) concentration, and the activity of various antioxidant enzymes such as superoxide dismutase (SOD), catalase (CAT), glutathione peroxidase (GSH-Px), and glutathione S-transferase (GST). The results revealed a significant decrease in plasma total antioxidant capacity and in the activity of CAT, GSH-Px, and SOD (*p* < 0.05) in the Wi-Fi-exposed group In contrast GST activity was significantly increased (*p* < 0.05). No significant changes were observed in GSH and TBARS levels. These findings suggest that antioxidant defense in rats exposed to Wi-Fi signals was markedly impaired compared with the control group, underscoring the need for further research to elucidate the biological mechanisms underlying the effects of electromagnetic radiation from Wi-Fi devices (17).

Kuybulu et al. (18) conducted a relevant study in this area, which investigated the effects of oxidative stress and apoptosis in renal tissues of male Wistar rats exposed to a Wireless electromagnetic field (EMF) with a frequency of 2.45 GHz for prolonged pre- and postnatal periods. Oxidative stress markers were assessed, and histological analysis of the renal tissues was performed. The results showed that malondialdehyde (MDA) and total oxidant (TOS) levels in the renal tissue of the prenatal group were elevated. In contrast, total antioxidant (TAS) and superoxide dismutase (SOD) levels were reduced. The spot urine N-acetyl-beta-Dglucosaminidase (NAG)/creatinine ratio was significantly higher in both the pre- and postnatal groups (*p* < 0.001). In addition, tubular injury was detected in most of the simples from the postnatal group. Immunohistochemical analysis revealed low intensity staining for Bax in the renal cortex and high intensity staining for Bcl-2 in the cortical and medullary areas in the prenatal group, with *p* values of 0.000, 0.002, and 0.000, respectively, compared with the control group. The Bcl-2/Bax staining intensity ratios in the medullary and cortical regions were significantly higher in the prenatal group than in the control group (*p* = 0.018 and *p* = 0.011, respectively). Kuybulu’s group concluded that chronic exposure to 2.45 GHz electromagnetic fields during the pre- and postnatal periods may induce chronic renal damage. They suggested that avoiding exposure to these frequencies, especially during pregnancy and early childhood, might mitigate the adverse effects on the kidneys.

The results of the Kuybulu research group indicate that elevated levels of malondialdehyde (MDA) and total oxidant (TOS), along with reduced levels of total antioxidant (TAS) and superoxide dismutase (SOD) in kidney tissue, indicate a situation of oxidative imbalance. It can be generally stated that during an oxidative process, reactive oxygen species (ROS), such as free radicals, are generated. These ROS have the potential to damage DNA, proteins, and cellular lipids, which can lead to mutations and contribute to the development of diseases, including cancer and leukemia. In this line, the research group led by Nazıroğlu et al. (19), demonstrated that oxidative stress and cell proliferation in leukemia (HL-60 cancer cells) can be induced by exposure to radiation from 2.45 GHz wireless devices. The results of their study concluded that these mobile or wireless devices, such as Wi-Fi, can affect biological systems by increasing the production of free radicals. Naziroğlu’s group found that the degree of lipid peroxidation, cytosolic free Ca^2+^ concentration, and cell number were significantly increased compared to controls when cells were irradiated with a 2.45 GHz WiFi source. Furthermore, the increase in cytosolic free Ca^2+^ concentration was dependent on the exposure time, peaking after 24 h of radiation. However, reduced glutathione, glutathione peroxidase, vitamin C, and cell viability values did not show significant changes in any of the experimental groups.

The relationship between Wi-Fi and temperature increases in body tissues has been investigated from both biological and physical perspectives. In particular, Christ’s group (20) has conducted studies showing the maximum temperature increase induced by electromagnetic fields in the range of 6 to 100 GHz. Using a stratified skin model, composed of four or five layers and exposed to plane waves generated by radio frequencies (RF), it was possible to identify that the maximum temperature increase observed was 0.4° C when applying the current power density limit for the general population, set at 10 W/m^2^. This skin model distinguishes the stratum corneum (SC) and the viable epidermis as the outermost layers. Despite these findings, Christ’s group suggests that directly investigating the temperature increase in higher biological systems is complex, due to the small temperature increases observed. In order to address this problem, Heat Shock Proteins (HSP) had to be taken into account. These proteins are produced in response to stress conditions, such as increased temperature, and help protect and repair other proteins that have become denatured or unstable due to stress. A small increase in temperature causes changes in expression levels and can be visualized using PCR techniques. In this context, Yang’s group investigated the effect of exposure to 2.45 GHz electromagnetic fields with a specific absorption rate (SAR) of 6 W/kg on the hippocampus of adult male Sprague- Dawley rats, whose bodies were immobilized. The objective was to evaluate stress by measuring the expression levels of heat shock proteins (HSPs). From a total of 2048 candidate genes analyzed, 23 upregulated genes and 18 downregulated genes were identified. Among these differentially expressed genes, two heat shock proteins stood out: HSP27 and HSP70, whose expressions were significantly increased in the hippocampus of exposed rats. Immunocytochemistry revealed enhanced staining of HSP27 and HSP70 in the hippocampus, especially in pyramidal neurons of cornu ammonis 3 (CA3) and granule cells of dentate gyrus (DG). The expression profiles of HSP27 and HSP70 were confirmed by reverse transcription polymerase chain reaction (RT-PCR) and Western blot. These results provide evidence that exposure to electromagnetic fields induces a stress response in the hippocampus of rats (21, 22).

Thanks to advancements in DNA microarray technologies, which enable the simultaneous analysis of thousands of genes in a relatively short period, Sakurai’s group investigated gene expression in a human glial cell line, SVGp12, exposed to continuous 2.45 GHz radiofrequency electromagnetic fields. Cells were exposed to specific absorption rates (SAR) of 1, 5, and 10 W/kg for 1, 4, and 24 h. Microarray analysis identified 23 mapped and 5 unassigned gene sites as potential genetic alterations. Of the 23 mapped gene sites, 22 were subsequently analyzed by reverse transcription-polymerase chain reaction (RT-PCR) to validate the microarray results. No significant alterations in gene expression were observed under the experimental conditions evaluated. The microarrays used contained 50 oligonucleotide probes for 30,000 genetic features. Of these, 10,000 genes were identified with names and functions, another 10,000 genes were identified by name, and the remaining 10,000 genes, selected as predicted expression genes by the laboratory, had neither name nor function assigned. The results showed groups of genes with reduced expression (FEN1, ANKRD36B, ANKRD57, SLC30A5, ADI1, PSPH, C1orf63, NAP5, ITM2B) and genes with increased expression (BTBD3, KLF16, BNIP3, EPHA8, RPL37A, NUP188, SLC25A1, TUBA1A, GNAI2, FARSA, HMG20B, CCDC86). However, Sakurai’s group concluded that no significant alterations in gene expression were found, suggesting that exposure to radiofrequency fields has no detectable effects on gene expression in SVGp12 cells (23).

Alzheimer’s disease (AD) is a progressive and complex neurodegenerative disease characterized by a strong genetic predisposition. This pathology initially manifests itself through difficulties in recent memory, evidenced by the inability to remember recent information or events. As the disease progresses, memory loss extends to past events, affecting the general ability to remember and retain information. Affected individuals also experience language difficulties, such as trouble finding the right words and following coherent conversations. In addition, notable changes in behavior are observed, where the person may become more irritable, confused, or suspicious. Habits and abilities to perform daily tasks also experience significant alterations. The incidence of the disease increases with age, especially after the age of 65. Concomitant health conditions, such as hypertension, diabetes and high cholesterol levels, have been associated with an increased risk of developing Alzheimer’s. Several genes have been identified as influential in the development and progression of the disease, including the APOE gene, which is associated with the risk of developing Alzheimer’s, located on chromosome 19. There are three main alleles: ε2, ε3, and ε4. The ε4 allele is associated with an increased risk of Alzheimer’s, while the ε2 allele may offer some protection (24). TREM2 (Triggering Receptor Expressed on Myeloid Cells 2) is involved in the function of microglial cells in the brain, located on chromosome 6 (25). CLU (Clusterin), The CLU gene is involved in the process of eliminating misfolded proteins and regulating inflammation in the brain, and is located on chromosome 8 (26). PICALM (Phosphatidylinositol Binding Clathrin-Associated Protein) is involved in the regulation of vesicle trafficking and cholesterol homeostasis, both important in the context of Alzheimer’s, is located on chromosome 11 (27). ABCA7 (ATP-Binding Cassette Subfamily A Member 7) is associated with Alzheimer’s risk and is involved in lipid transport and cholesterol metabolism, located on chromosome 19 (28). SORL1 (Sortilin-Related Receptor 1), involved in APP trafficking and *β*-amyloid metabolism, located on chromosome 11 (29). GSK3B (Glycogen Synthase Kinase 3 Beta), is involved in the phosphorylation of TAU, a protein that accumulates in neurons affected by Alzheimer’s, located on chromosome 3 (30). PRNP (Prion Protein), best known for its association with Creutzfeldt-Jakob disease, has been implicated in some forms of Alzheimer’s disease and is located on chromosome 20 (31).

Currently, a variety of genes have been identified that play a role in regulating Alzheimer’s disease. In this review, we will focus specifically on risk genes associated with Alzheimer’s disease that are not linked to hereditary forms. This study aims to investigate how exposure to 2.4 GHz Wi-Fi radiation might influence genes such as ABCA7, PICALM, CLU, TREM2, APOE, SORL1, GSK3B and PRNP, which are considered risk factors for the disease. To address this question, we will explore the STRING database to determine possible relationships between these genes and heat shock proteinsHSP27 or HSPB1 and HSP70 or HSPA4. Additionally, we will examine whether there is any association between the mentioned genes and other genes with differential expressions such as FEN1, ANKRD36B, ANKRD57, SLC30A5, ADI1, PSPH, C1orf63, NAP5, ITM2B, BTBD3, KLF16, BNIP3, EPHA8, RPL37A, NUP188, SLC25A1, TUBA1A, GNAI2, FARSA, HMG20B and CCDC86.

## Materials and methods

**Figure 1:**
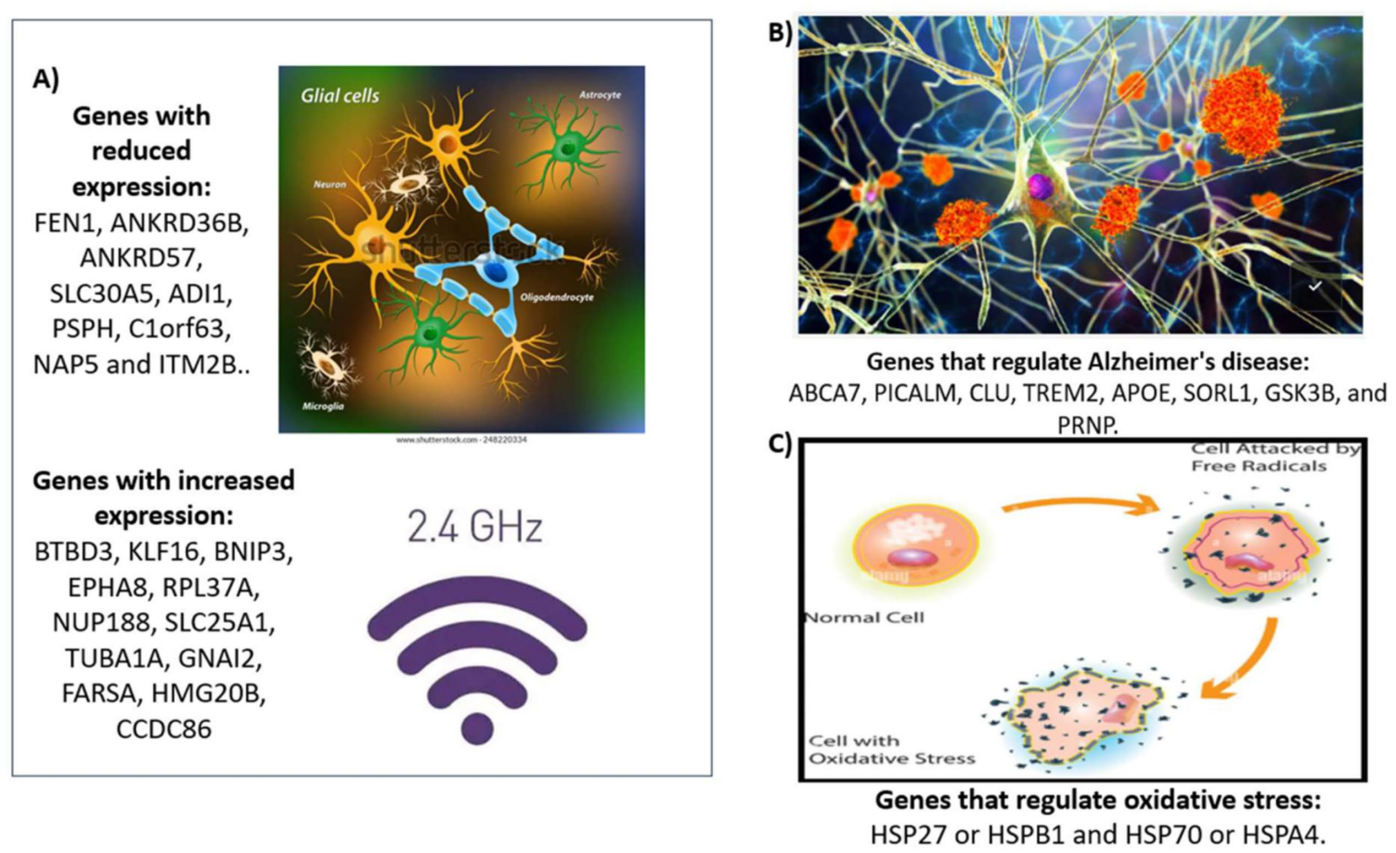
Methodological plan of review study. The image represents three panels, **(A)** represents the expression levels of the increased and reduced genes when presented by Sakurai et al. (23). **(B)** represents the genes that regulate Alzheimer’s disease and **(C)** represents the genes that regulate oxidative stress.

The present review article is based on a comprehensive and systematic collection of relevant data aimed at evaluating the health effects of mobile phone usage and associated telecommunications technologies. A structured methodology was developed, consisting of the following phases (see Figure 1).

This review was conducted following the guidelines of the PRISMA statement (Preferred Reporting Items for Systematic Reviews and Meta-Analyses) and unified Systematic Review Matrix: Oxidative Stress in Alzheimer’s and from Wi-Fi Exposure (see Supplementary Table 1), with the aim of systematically and comprehensively collecting scientific literature focused on evaluating health effects associated with the use of mobile phones and related telecommunication technologies. This methodological approach ensures compliance with the requirements for transparency, replicability, and methodological quality in the processes of selecting and analyzing scientific literature. In addition, research questions were formulated and applied to guide the search and selection strategy, with the purpose of including in the review only those articles that directly address the stated questions.

### Research questions

Q1: How does exposure to 2.4 GHz electromagnetic fields affect gene expression related to oxidative stress response, genomic stability, metabolism, and cellular regulation?

Q2: How does the regulation of oxidative stress and protein homeostasis influence the progression of neurodegeneration in Alzheimer’s disease?

Q3: Can genes associated with Alzheimer’s disease functionally interact with genes involved in DNA repair, metabolism, and cellular regulation?

The literature search strategy was conducted in mid-2024, following the PICOC approach (population, intervention, comparison, outcomes, context) (see Supplementary Table 2). The study population included animal models (rats), human cell lines, and tissues used to investigate the biological impacts of Wi-Fi radiation exposure. The intervention corresponded to exposure to 2.4 GHz non-ionizing electromagnetic radiation emitted by Wi-Fi devices. As for the comparisons, organisms and systems not exposed to Wi-Fi or to other sources of electromagnetic radiation or related frequencies were considered. The outcomes of interest included changes in oxidative stress levels, gene expression, cellular damage biomarkers, and associations with neurodegenerative processes such as Alzheimer’s disease. The context of this review was limited to scientific literature published between 1992 and 2024, within the fields of biomedical, technological, and bioinformatics research.

### Study selection and categorization

Original research articles, as well as relevant systematic reviews and meta-analyses, were included in this review. The inclusion criteria were restricted to publications written in English, available in peer-reviewed, indexed journals, and with full-text access. The literature search covered studies published between 1992 and 2024. Exclusion criteria comprised articles without full-text access, duplicates, and publications lacking a direct focus on the biological effects of electromagnetic radiation. This selection strategy ensured the inclusion of current and thematically relevant scientific literature in line with the objectives of the present review.

The included studies evaluated the impact of electromagnetic radiation on biological, histopathological, and molecular parameters. Both *in vitro* and *in vivo* models with clearly defined experimental designs were considered eligible for inclusion.

Selected studies were thematically categorized into three groups:

- Molecular evaluations, including effects on oxidative stress biomarkers, genetic damage, and gene expression;
- Histopathological changes in tissues, focusing on the reproductive, cardiovascular, renal, and nervous systems;
- Epidemiological studies and international regulatory guidelines regarding exposure to electromagnetic fields (EMF).

### Inclusion and exclusion criteria

IC1: Original studies, systematic reviews, and meta-analyses.

IC2: Articles with full-text access.

IC3: Studies published in English.

IC4: Scientific publications corresponding to the period 1992–2024.

IC5: Publications in indexed journals.

IC6: Research addressing at least one of the following topics:

- Molecular evaluations
- Histopathological changes in tissues
- Epidemiological studies and international regulatory guidelines on EMF exposure

EC1: Studies without full-text access.

EC2: Duplicate articles, opinion pieces, letters to the editor.

EC4: Publications lacking a direct focus on the biological effects of electromagnetic radiation.

### Search strategy

The data sources used were the Scopus, PubMed, Web of Science, and Google Scholar databases, selected for their broad multidisciplinary coverage and inclusion of peer-reviewed scientific literature. To identify relevant studies, the following Boolean operators were applied:

“2.45 GHz electromagnetic fields,” “health effects of Wi-Fi exposure,” “radiofrequency radiation and oxidative stress,” “gene expression under electromagnetic fields,” and “biological impacts of broadband technologies.”

Subsequently, the articles indexed in the databases were exported, processed, and organized in spreadsheets for further analysis.

### Study selection

**Figure 2:**
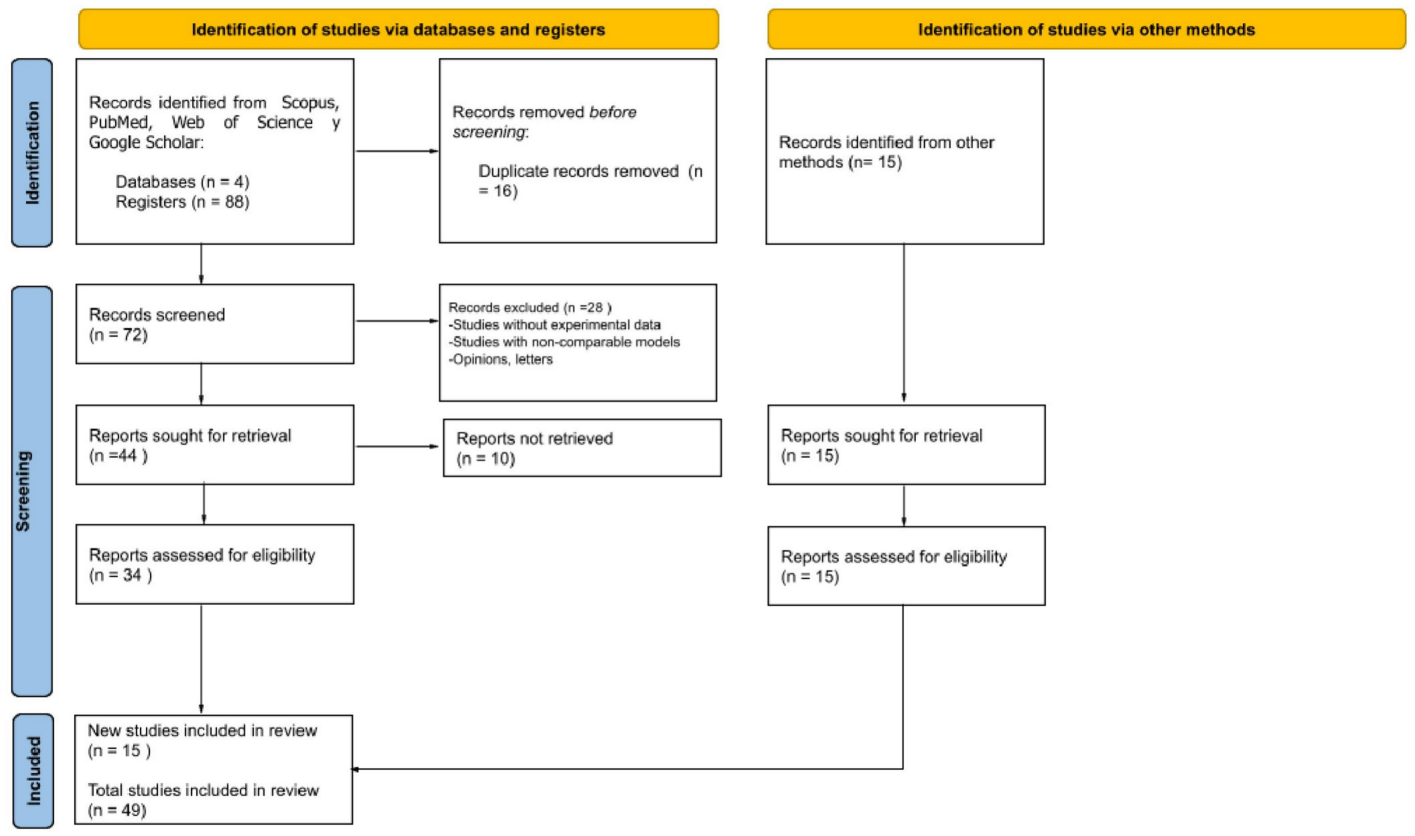
PRISMA 2020 flow diagram for new systematic reviews which included searches of databases, registers and other sources.

The initial search yielded a total of 103 articles. After applying the research questions and key terms (keywords), a filtering process was conducted to select the most relevant studies. The selection process was carried out in two stages. The first stage consisted of an initial screening by reviewing titles and abstracts, excluding studies that did not meet the previously established eligibility criteria. In the second stage, the preselected articles were analyzed in full text to determine their final inclusion in the review. A total of 49 studies were selected and included in the systematic review. The entire selection procedure followed the PRISMA flow diagram, indicating the number of documents identified, screened, excluded, and included in the review (see Figure 2).

After completing the study selection process, systematic data extraction was conducted. A structured and standardized spreadsheet was used to record the following: study authors, year of publication, abstract, type of model used (*in vivo* or *in vitro*), parameters evaluated (biomarkers, gene expression, histological alterations, among others), applied methodology, and key findings. Additionally, articles were organized according to their corresponding thematic category. The objective of the comprehensive data extraction was to identify recurring patterns, gaps in the literature, relevant findings, and to answer the proposed research questions. Moreover, the aim was to establish links between exposure to telecommunication technologies and potential adverse health effects. To ensure proper organization and management of bibliographic references, Mendeley software was used.

For the analysis of molecular and genetic data, techniques such as DNA microarrays, RT-PCR, Western blot, and ELISA were employed. In histological studies, parameters such as tissue volumetric density, antioxidant levels, cellular damage biomarkers, and the expression of heat shock proteins (HSP27 and HSP70) were evaluated. Additionally, for protein–protein interaction studies and the assessment of possible alterations in signaling networks associated with the effects of electromagnetic radiation, the STRING database (32) was used. This tool played a key role in identifying known and predicted protein interactions, enabling a deeper analysis of how proteins affected by electromagnetic fields may be involved in biological processes including oxidative stress, gene expression, and cellular repair.

### Ethical and regulatory criteria

Despite being a systematic review, this work only included studies that explicitly reported compliance with current international ethical guidelines for animal experimentation and research involving human cell lines were selected.

## Results

### Results of study selection (PRISMA 2020)

A total of 103 records were initially identified through database searching and other sources. After removing 16 duplicates, 72 records remained and were screened based on title and abstract. Following this screening process, 28 records were excluded for not meeting the inclusion criteria. The full text of the remaining 34 articles was assessed for eligibility. However, 10 full-text articles could not be retrieved, despite attempts to access them. No studies were excluded after full-text review. Ultimately, 15 new studies meeting the eligibility criteria were included in the review. Additionally, 34 studies from previous selections were retained. Therefore, a total of 49 studies were included in the final synthesis (see Figure 2) (see Supplementary Table 4).

### Risk genes in Alzheimer’s disease

In the last decade, advances in genetic research have enabled the identification of multiple genes associated with an increased risk of developing Alzheimer’s disease (AD). This review analyzes eight key genes that, while not linked to hereditary forms of the disease, significantly contribute to its pathogenesis. The analysis highlights how specific genetic variants influence the main pathological processes of AD, such as beta-amyloid accumulation, synaptic dysfunction, and inflammation. Identifying these genes not only deepens our understanding of the molecular mechanisms underlying AD but also opens avenues for developing new therapeutic strategies aimed at slowing its progression.

**Figure 3:**
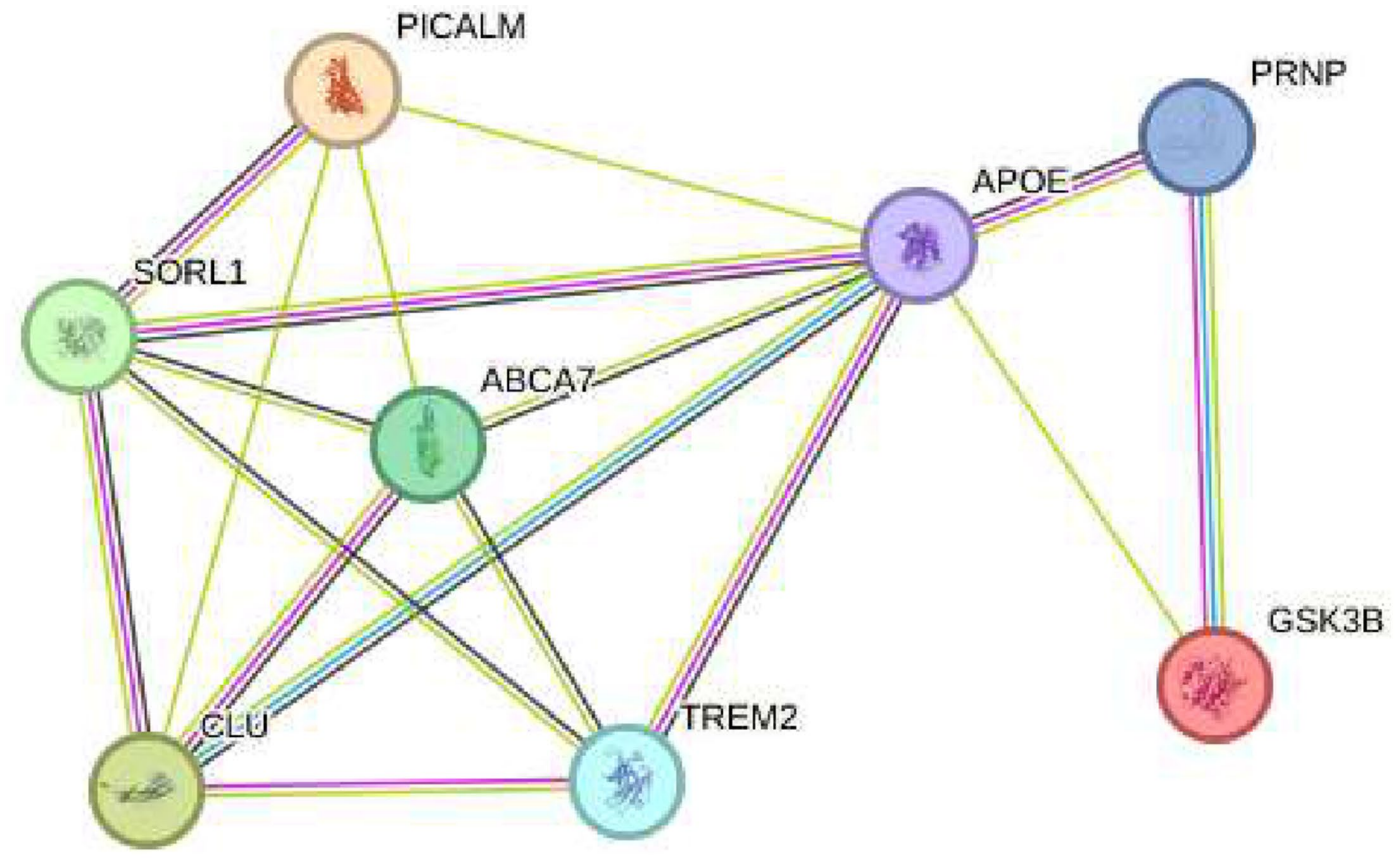
Risk genes in Alzheimer’s disease: various non hereditary genetic variants, such as ABCA7, PICALM, CLU, TREM2, APOE, SORL1, GSK3B, and PRNP, contribute to beta-amyloid accumulation, synaptic dysfunction, and inflammation in Alzheimer’s disease.

The first gene discussed is ABCA7, which encodes a lipid transporter crucial for regulating lipid metabolism and clearing beta-amyloid protein. Variants in ABCA7 have been associated with an increased risk of beta-amyloid accumulation, a primary neuropathological hallmark of AD (33). In contrast, PICALM plays a role in vesicle formation and trafficking at neuronal synapses, essential processes for maintaining neuronal homeostasis. Variants in PICALM disrupt protein recycling at the synapse, worsening synaptic dysfunction observed in AD (34). Similarly, the CLU (Clusterin) gene contributes to beta-amyloid clearance through its function as an extracellular apolipoprotein. Variants in CLU reduce amyloid clearance capacity, thereby promoting its accumulation in the brain (35). Likewise, TREM2 encodes a receptor expressed in microglial cells that is essential for the brain’s inflammatory response. Mutations in TREM2 impair microglial ability to clear beta-amyloid and control inflammation, exacerbating neuronal damage (36). The APOE gene is the most well-known genetic risk factor for late-onset AD, particularly its ε4 allele. APOE participates in lipid transport and neuronal repair, and the ε4 variant is associated with increased beta-amyloid accumulation (37). Additionally, SORL1, which regulates intracellular trafficking of the amyloid precursor protein (APP), has variants that alter APP processing pathways, promoting beta-amyloid production and increasing the pathological burden (38). Furthermore, GSK3B (glycogen synthase kinase 3 beta) is involved in tau protein hyperphosphorylation, an event that leads to the formation of neurofibrillary tangles. Dysfunctional GSK3B activity is linked to accelerated neurodegeneration in AD (39). Lastly, although the PRNP gene is primarily associated with prion diseases, it has been suggested to interact with beta-amyloid, modulating its toxicity and indirectly contributing to AD pathogenesis (40) (see Figure 3).

### Implications for protection against cellular damage

**Figure 4:**
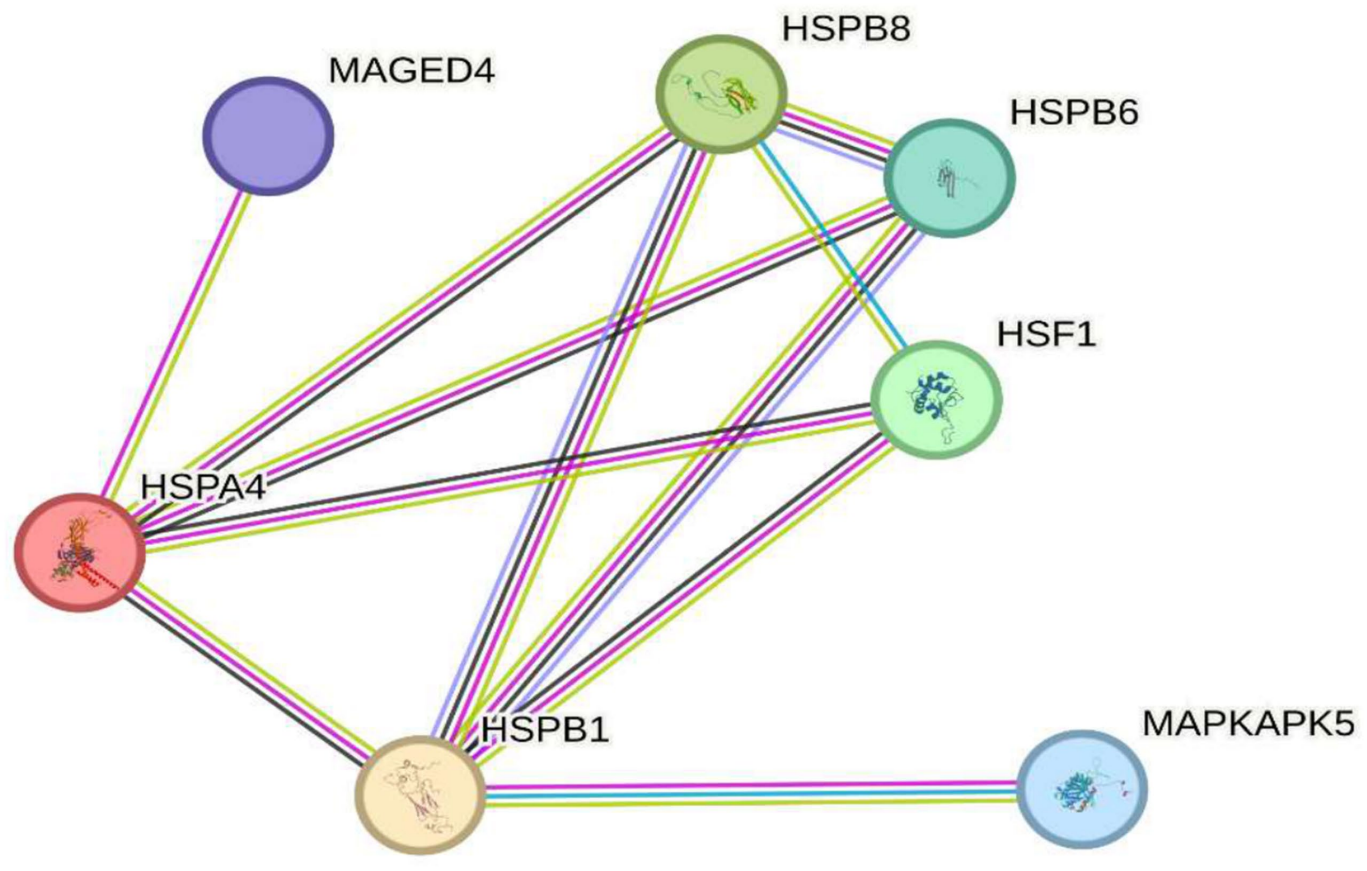
Implications in protection against cellular damage. The image shows seven key genes (MAGED4, HSPB8, HSPB6, HSF1, HSPA4, HSPB1, and MAPKAPK5) involved in the cellular response to oxidative stress. These genes regulate protein homeostasis, stabilize misfolded proteins, and protect against apoptosis induced by reactive oxygen species (ROS), highlighting their role in preventing cellular damage and diseases associated with oxidative stress.

It has been observed that the genes MAGED4, HSPB8, HSPB6, HSF1, HSPA4, HSPB1, and MAPKAPK5 play crucial roles in the cellular response to oxidative stress, a key factor in the pathogenesis of various diseases. These genes contribute to cellular protection by regulating protein homeostasis, stabilizing misfolded proteins, and preventing apoptosis induced by reactive oxygen species (ROS), underscoring their importance in preventing oxidative stress related disorders. In this regard, Figure 4 depicts circles representing these seven genes and their functional connections to oxidative stress. Among them, MAGED4 has been identified as a key regulator of apoptosis and the cellular response to oxidative stress induced damage. Specifically, MAGED4 appears to mediate protection against ROS, promoting cellular repair and reducing oxidative injury (6), and its role in responding to cellular damage suggests it is essential for defense under prolonged stress conditions. Similarly, HSPB8, a heat shock protein, plays a fundamental role in protecting against oxidative stress by acting as a molecular chaperone that prevents the accumulation of misfolded proteins, a critical defense against ROS-induced cellular damage and it also interacts with various oxidative stress related proteins, contributing to cellular homeostasis (41). Another heat shock protein, HSPB6, stabilizes proteins and membranes exposed to oxidative damage, and its overexpression enhances resistance to oxidative stress, thereby reducing ROS induced apoptosis (42). In addition, HSF1 (heat shock factor 1) is the primary transcriptional regulator of stress response, inducing the expression of protective genes such as heat shock proteins; its activation by ROS triggers the production of proteins that defend cells from oxidative damage, highlighting its pivotal role in maintaining protein homeostasis under oxidative conditions (43). Complementing this, HSPA4, another stress induced heat shock protein, protects against ROS-mediated accumulation of misfolded proteins by facilitating protein refolding, thereby preserving cellular function (44). Moreover, HSPB1, well known for its role in thermal stress protection, is also important in responding to oxidative stress as it stabilizes proteins and membranes damaged by ROS and regulates apoptosis, contributing to protection against severe oxidative injury (45). Finally, MAPKAPK5, a MAPK-activated kinase, modulates the cellular response to oxidative stress by inducing transcription of protective genes and participating in inflammation regulation, further shielding cells from ROS induced damage (46).

On the other hand, exposure to 2.4 GHz electromagnetic fields (EMF) has been shown to alter the expression of several genes associated with DNA replication, cellular homeostasis, and metabolism. Among these, POLD4 and FEN1, both essential for DNA replication and repair, show altered expression that could compromise genomic stability. Additionally, EXOG, involved in mitochondrial DNA repair, exhibits changes suggesting potential disruption of mitochondrial function. Similarly, ITM2B, related to apoptosis, and NAP5, which participates in chromatin organization, display expression variations that may impact gene regulation and programmed cell death. Regarding metabolism, genes such as PSPH and ADI1, involved in amino acid biosynthesis, also undergo modifications, indicating possible imbalances in cellular homeostasis. Moreover, zinc transporters like SLC30A5 and SLC39A9 respond to this radiation, potentially affecting the availability of this essential metal critical for various biological processes. Furthermore, genes involved in epigenetic and transcriptional regulation such as ENOPH1, RSRP1, and SOWAHC exhibit expression changes that may influence cellular signaling pathways. Lastly, ANKRD36B, encoding a protein with ankyrin repeat domains involved in protein–protein interactions and cytoskeleton organization, is also affected.

**Figure 5:**
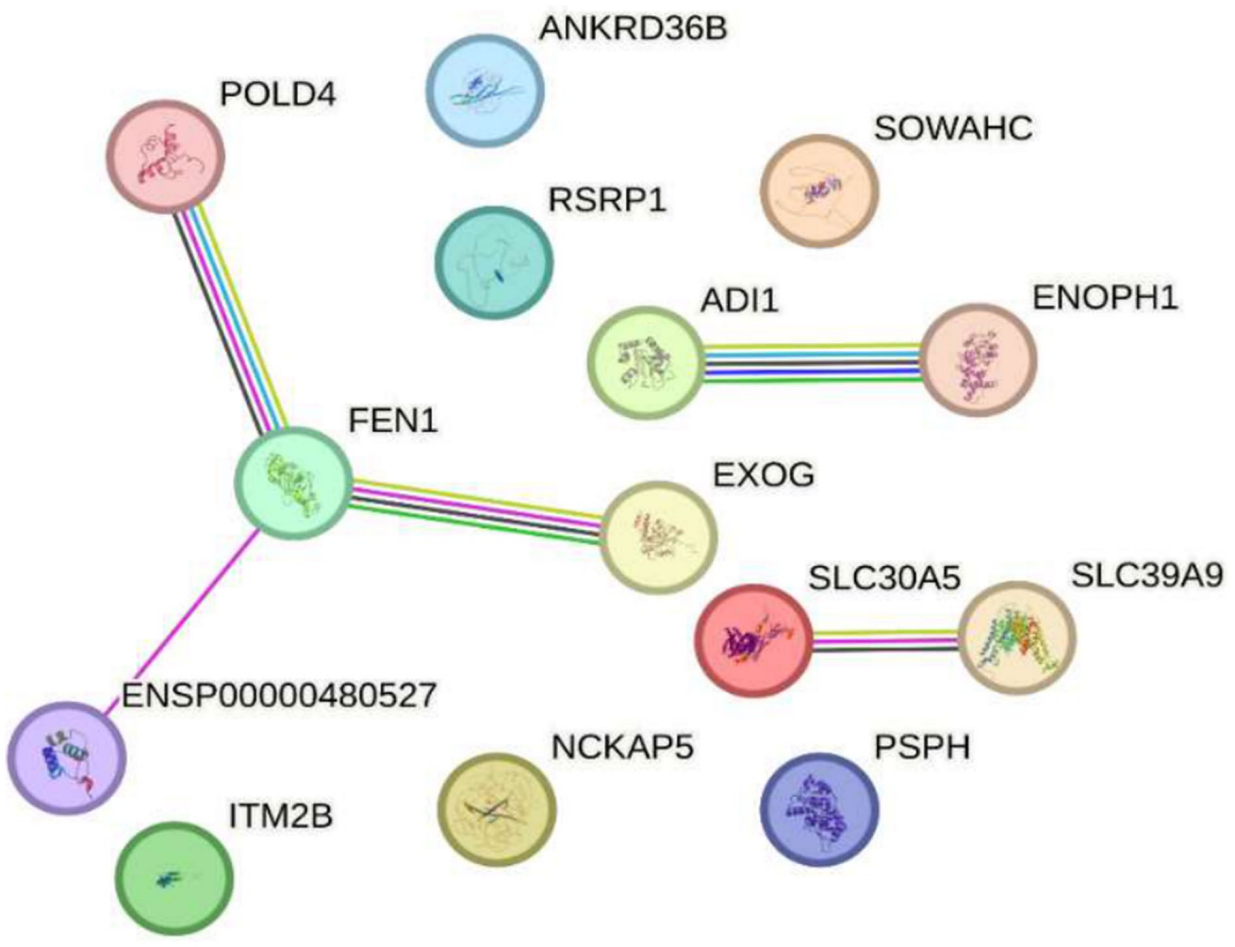
Impact of exposure to 2.4 GHz electromagnetic fields on reduced gene expression Sakurai et al. (23). The image shows interconnected genes responding to 2.4 GHz electromagnetic field exposure from Wi-Fi devices. Genes with altered expression were identified, participating in key processes like DNA replication, cellular homeostasis, and metabolism. Notable examples include POLD4 and FEN1 (DNA repair), EXOG (mitochondrial repair), ITM2B (apoptosis), NAPS (chromatin regulation), PSPH and ADI1 (metabolism), as well as zinc transporters and genes related to epigenetic and transcriptional regulation. These changes suggest that 2.4 GHz radiation may affect genomic stability, metabolism, and cellular regulation, highlighting the need for further research on its biological effects.

Together, these findings suggest that exposure to 2.4 GHz EMF can significantly alter genomic stability, metabolism, and cellular regulation. In this context, Figure 5 shows circles representing genes connected by lines of different colors indicating various types of interactions. The gene distribution, obtained from the STRING database, reflects the response to 2.4 GHz EMF emitted by Wi-Fi devices, which have been studied for their potential impact on gene expression and cellular regulation. A set of genes has been identified whose expression is altered by this radiation, implicating key biological processes such as DNA replication, cellular homeostasis, and metabolism. For example, POLD4 encodes an accessory subunit of DNA polymerase delta essential for DNA replication and repair; exposure to 2.4 GHz EMF has been reported to affect its expression, possibly compromising genomic stability (6). Similarly, FEN1, an endonuclease key to Okazaki fragment maturation and DNA repair, shows altered expression that could influence DNA integrity (42). EXOG, involved in mitochondrial DNA repair, also displays expression changes after EMF exposure, suggesting mitochondrial dysfunction and impaired energy metabolism (41). Likewise, ITM2B, which regulates apoptosis and proteolysis, is affected, potentially impacting programmed cell death (43). Genes related to chromatin regulation, such as NAP5, which participates in nucleosome assembly, show altered expression that might disrupt gene regulation and chromatin structure (6). Metabolic genes like PSPH, involved in serine biosynthesis, and ADI1, regulating methionine metabolism, also respond to 2.4 GHz EMF exposure, indicating disruptions in essential pathways for cellular homeostasis (41). Zinc transporters SLC30A5 and SLC39A9 react similarly, suggesting altered zinc homeostasis (42). Additionally, ENOPH1, which participates in methylation and methionine biosynthesis, is affected, with possible epigenetic implications (43). Regarding transcriptional regulation, RSRP1 (C1orf63) and SOWAHC (ANKRD57) exhibit expression modifications potentially influencing gene regulation and signal transduction. Finally, ANKRD36B, encoding a protein with ankyrin repeat domains involved in protein–protein interactions and cytoskeletal dynamics, is also altered (6). Overall, these results highlight the need to further investigate the effects of 2.4 GHz EMF exposure on gene expression due to its possible impact on genomic stability, metabolism, and molecular homeostasis (see Figure 5).

**Figure 6:**
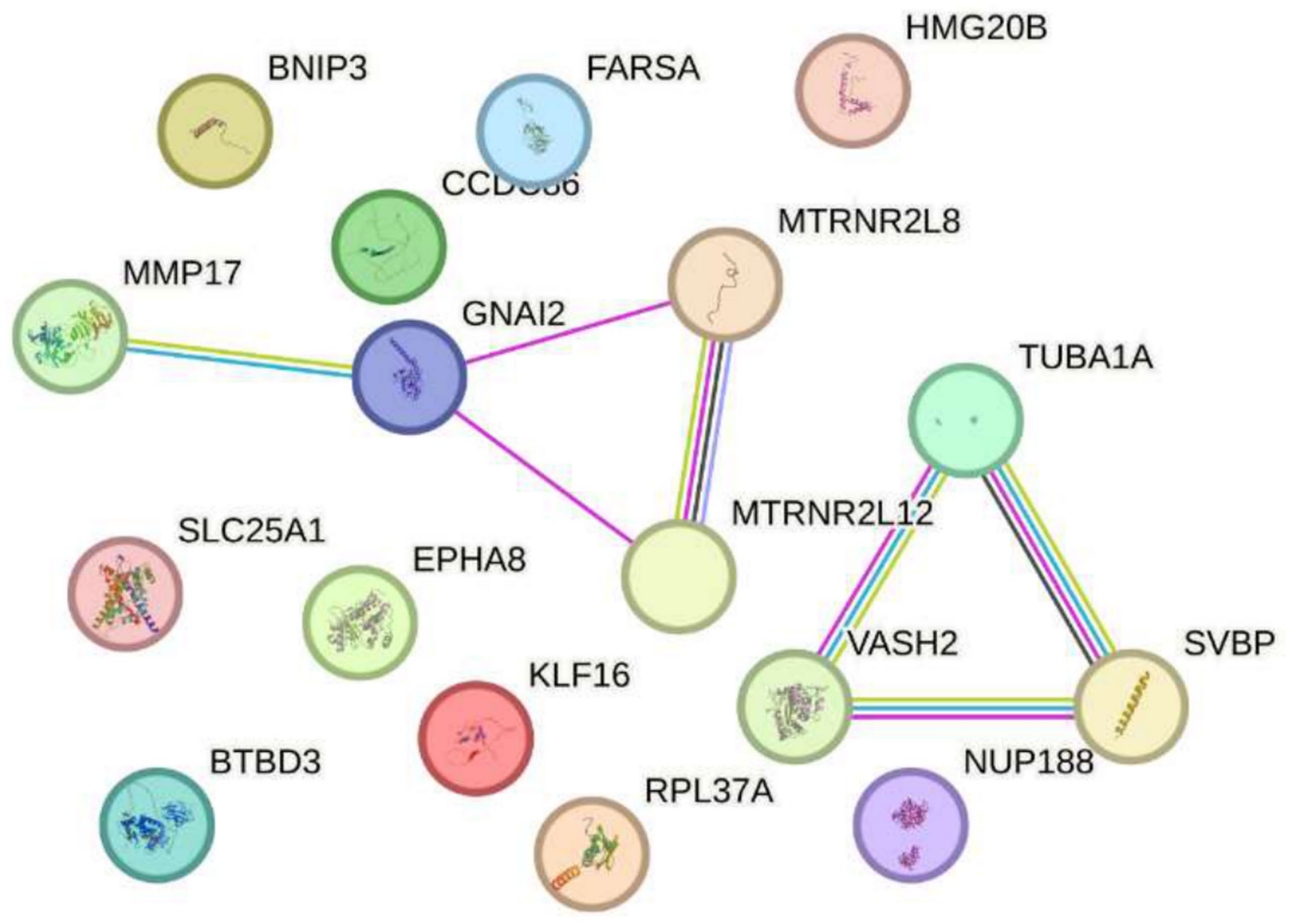
Impact of exposure to 2.4 GHz electromagnetic fields on increased gene expression Sakurai et al. (23). No direct evidence has been found linking the genes slc25a1, btbd3, epha8, klf16, among others, to electromagnetic radiation at 2.4 GHz or other frequencies. Research in this field is still emerging, and most studies have not shown significant changes in gene expression. Organizations such as the WHO continue to monitor the evidence to guide scientific recommendations.

Finally, after a thorough review of the available scientific literature, no studies have demonstrated a direct response of the genes SLC25A1, BTBD3, EPHA8, KLF16, RPL37A, NUP188, FARSA, HMG20B, CCDC86, BNIP3, MMP17, GNAI2, MTRNR2L8, MTRNR2L12, VASH2, TUBA1A, and SVBP to electromagnetic radiation at either 2.4 GHz or other frequencies. Research on the effects of electromagnetic radiation on gene expression is an emerging field, and currently, there is no evidence specifically linking these genes to such exposure. It is important to note that most studies to date have not identified significant changes in gene expression due to electromagnetic radiation. Organizations such as the World Health Organization (WHO) continue to monitor research in this area to provide guidelines based on scientific evidence (see Figure 6).

Genes that regulate Alzheimer’s disease ABCA7, PICALM, CLU, TREM2, APOE, SORL1, GSK3B, and PRNP crossed with the genes HSP27 or HSPB1 and HSP70 or HSPA4 that regulate oxidative stress.

According to the reviewed literature, glycogen synthase kinase 3 beta (GSK3B) is a key serine/threonine kinase involved in multiple cellular pathways, including energy metabolism regulation, embryonic development, and neurogenesis. In Alzheimer’s disease (AD), GSK3B plays a critical role in phosphorylating tau protein, contributing to the formation of neurofibrillary tangles, a hallmark of the disease. Additionally, GSK3B regulates the cleavage of amyloid precursor protein (APP), which leads to the production of *β*-amyloid (Aβ), another characteristic marker of AD. Overactivation of GSK3B is further associated with neuroinflammation and oxidative damage, processes that exacerbate neurodegeneration in AD (47).

Building upon this, heat shock proteins (HSPs) 27 (HSPB1) and 70 (HSPA4) act as molecular chaperones, protecting cells from proteotoxic stress by facilitating proper protein folding and preventing aggregation. In AD, overexpression of HSP27 has been observed to improve neuronal excitability and reduce Aβ plaque formation in animal models, suggesting a neuroprotective role (48). Similarly, HSP70 has demonstrated potential to suppress AD progression in both *in vitro* and *in vivo* studies, positioning it as a promising therapeutic target (49).

Importantly, the interaction between GSK3B and these HSPs centers on the regulation of oxidative stress and the maintenance of protein homeostasis. GSK3B can phosphorylate and modulate the activity of HSPs, thereby influencing their ability to prevent protein misfolding and aggregate formation. Consequently, changes in the expression levels of GSK3B or HSPs may impair the cell’s capacity to manage oxidative stress, worsening neurodegeneration in AD. Specifically, overexpression of GSK3B may increase tau phosphorylation and Aβ production, intensifying oxidative stress and neuronal inflammation. Conversely, reduced GSK3B expression could lower tau phosphorylation and Aβ formation but might also negatively affect other essential cellular functions regulated by GSK3B, such as neurogenesis and synaptic plasticity.

Regarding HSPs, elevated levels of HSP27 or HSP70 can enhance cellular defenses against oxidative and proteotoxic stress, thereby providing neuroprotection. However, excessive overexpression might disrupt normal cellular processes like apoptosis and autophagy, which are vital for clearing damaged proteins. On the other hand, decreased expression of HSP27 or HSP70 could weaken the cell’s stress response, increase neuronal vulnerability and accelerating AD progression.

**Figure 7:**
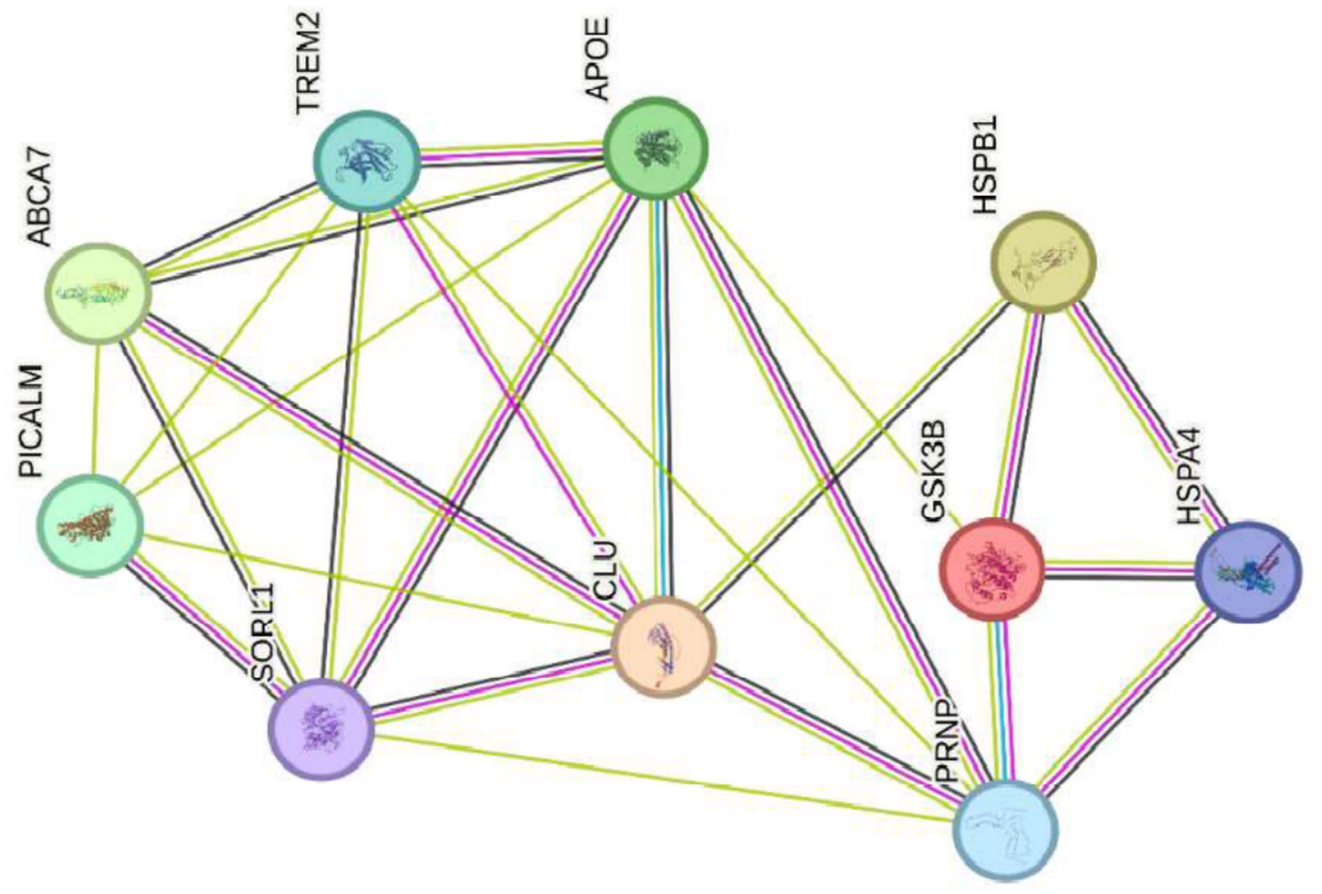
Genes that regulate Alzheimer’s disease ABCA7, PICALM, CLU, TREM2, APOE, SORL1, GSK3B, and PRNP crossed with the genes HSP27 or HSPB1 and HSP7Q HSPA4 that regulate oxidative stress.

In summary, precise regulation of GSK3B and HSPs (HSPB1 and HSPA4) is essential for maintaining protein and neuronal homeostasis (see Figure 7). Therefore, dysregulation of these genes compromises the cell’s ability to handle oxidative and proteotoxic stress, contributing significantly to the neurodegeneration observed in Alzheimer’s disease.

Genes that regulate Alzheimer’s disease ABCA7, PICALM, CLU, TREM2, APOE, SORL1, GSK3B, and PRNP crossed with genes with reduced expression at 2.4 GHz.

**Figure 8:**
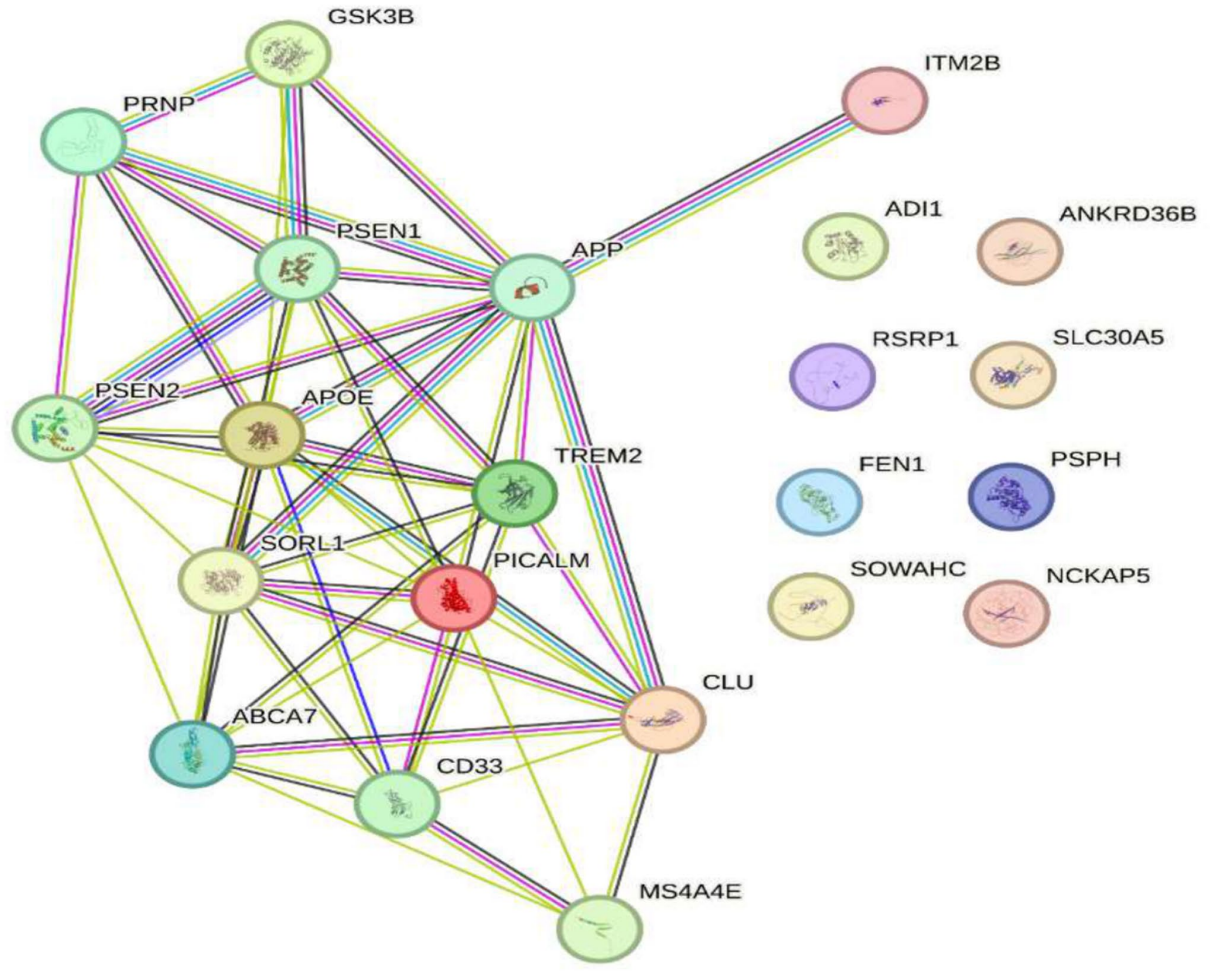
Genes that regulate Alzheimer’s disease ABCA7, PICALM, CLU, TREM2, APOE, SORL1, GSK3B, and PRNP crossed with the genes of reduced expression to 2.4 GHz. No studies have been found that establish a direct relationship between Alzheimer’s disease-associated genes (ABCA7, PICALM, CLU, TREM2, APOE, SORL1, GSK3B, PRNP) and genes POLD4, FEN1, EXOG, ITM2B, NAP5, PSPH, ADI1, SLC30A5, SLC39A9, ENOPH1, RSRP1, SOWAHC, ANKRD36B. However, some genes like GSK3B and APOE are involved in cellular processes that could influence the function of other genes. Further research is needed to understand possible interactions.

Regarding the relationship between genes associated with Alzheimer’s disease (ABCA7, PICALM, CLU, TREM2, APOE, SORL1, GSK3B, and PRNP) and the genes POLD4, FEN1, EXOG, ITM2B, NAP5, PSPH, ADI1, SLC30A5, SLC39A9, ENOPH1, RSRP1, SOWAHC, and ANKRD36B, no studies have established a direct link between these two groups. Research to date has primarily focused on genes related to Alzheimer’s disease, while the latter group is mainly involved in fundamental cellular functions such as DNA replication, DNA repair, and cellular homeostasis. However, some Alzheimer’s-related genes, such as APOE and GSK3B, participate in cellular processes that could potentially influence the function of other genes. For instance, GSK3B regulates multiple cellular pathways, including energy metabolism, embryonic development, and neurogenesis. As previously mentioned, in Alzheimer’s disease, GSK3B contributes to tau protein phosphorylation, promoting the formation of neurofibrillary tangles a hallmark pathological feature of the disease. Moreover, GSK3B modulates the cleavage of amyloid precursor protein (APP), resulting in the formation of *β*-amyloid (Aβ), another characteristic marker of Alzheimer’s disease. Overactivation of GSK3B is also linked to neuroinflammation and oxidative damage, processes that aggravate neurodegeneration in Alzheimer’s disease (50). On the other hand, heat shock proteins (HSPs) 27 (HSPB1) and 70 (HSPA4) serve as molecular chaperones that protect cells from proteotoxic stress by facilitating proper protein folding and preventing the formation of protein aggregates. In Alzheimer’s disease, overexpression of HSP27 improves neuronal excitability and reduces Aβ plaque formation in animal models, suggesting a neuroprotective effect. Likewise, HSP70 has demonstrated potential to suppress Alzheimer’s progression in both *in vitro* and *in vivo* studies, making it a promising therapeutic target (50). Although no direct associations have been established between Alzheimer’s disease genes and those with altered expression under 2.4 GHz exposure, there may be indirect interactions via shared cellular pathways or genetic regulatory mechanisms. Nonetheless, further research is needed to elucidate these potential interactions and their implications for Alzheimer’s disease pathogenesis (see Figure 8).

## Discussion

Recent research on Alzheimer’s disease (AD) has identified multiple genes that contribute to its pathogenesis. Among these are ABCA7, PICALM, CLU, TREM2, APOE, SORL1, GSK3B, and PRNP (see Figure 3), which are not associated with hereditary forms of the disease but play a crucial role in the accumulation of *β*-amyloid proteins, synaptic dysfunction, and neuroinflammation (35, 51). These genes are found in a highly dynamic biological environment, where oxidative stress plays a central role in the cellular damage associated with neurodegeneration (36). In particular, the activation of heat shock proteins such as HSPB1 and HSPA4 (see Figure 4), which protect cells from oxidative stress, is of particular interest in Alzheimer’s disease, as they could counteract the harmful effects of free radicals and misfolded proteins. Oxidative stress is a common feature in various neurodegenerative diseases, and the activation of response pathways protects cells against damage induced by reactive oxygen species (ROS) (43). In the context of AD, oxidative stress contributes to the accumulation of β-amyloid and the hyperphosphorylation of the tau protein, two of the most significant markers of the disease (39). Genes involved in the oxidative stress response, such as MAGED4, HSPB8, HSPB6, and HSF1, play a protective role by stabilizing misfolded proteins and regulating apoptosis induced by ROS, suggesting that their alteration could accelerate disease progression (46). The exposure to electromagnetic radiation, particularly at 2.4 GHz frequencies emitted by Wi-Fi devices, has been the subject of debate regarding its potential impact on cellular health. Several studies suggest that exposure to 2.4 GHz electromagnetic fields (EMF) could alter the expression of genes involved in cellular homeostasis, DNA repair, and metabolism (23). Among the affected genes are those like POLD4, FEN1, EXOG, and ITM2B, which are involved in DNA replication and repair processes, suggesting that exposure to this radiation frequency could compromise genomic stability and mitochondrial function. Our findings have not identified studies that demonstrate a direct relationship between electromagnetic radiation, whether at 2.4 GHz or other frequencies, and the activation or alteration in the expression of the genes slc25a1, btbd3, epha8, klf16, rpl37a, nup188, farsa, hmg20b, ccdc86, bnip3, mmp17, gnai2, mtrnr2l8, mtrnr2l12, vash2, tuba1a, and svbp (see Figure 6). If the electromagnetic radiation generated by 2.4 GHz Wi-Fi affects the expression of these genes, it could induce cellular damage and promote neuronal dysfunction, which in turn could contribute to the development of neurodegenerative diseases such as Alzheimer’s. One result we expected to find is that genes related to Alzheimer’s disease, such as ABCA7, PICALM, CLU, TREM2, APOE, SORL1, GSK3B, and PRNP, interact with genes such as HSP27 or HSPB1 and HSP70 or HSPA4, which are key in regulating oxidative stress. Figure 7 illustrates the functional connection between GSK3B, HSPB1, and HSPA4. According to various sources, it has been determined that GSK3B (glycogen synthase kinase 3 beta) is a serine/threonine kinase essential in several cellular processes, such as regulating energy metabolism, embryonic development, and neurogenesis. In the context of Alzheimer’s disease (AD), GSK3B is involved in the phosphorylation of tau protein, a mechanism that facilitates the formation of neurofibrillary tangles, one of the most characteristic pathological signs of the disease. In relation to our findings, the genes involved in the regulation of Alzheimer’s disease, such as ABCA7, PICALM, CLU, TREM2, APOE, SORL1, GSK3B, and PRNP, were intersected with the genes POLD4, FEN1, EXOG, ITM2B, NAP5, PSPH, ADI1, SLC30A5, SLC39A9, ENOPH1, RSRP1, SOWAHC, and ANKRD36B, which showed reduced expression after exposure to 2.4 GHz Wi-Fi. However, no studies have been found that establish a direct relationship between these groups of genes (see Figure 8). Although no studies have demonstrated a direct relationship between Alzheimer’s genes and those affected by 2.4 GHz EMF, the possibility that exposure to this radiation might indirectly influence the expression of key genes like APOE and GSK3B cannot be ruled out. As mentioned earlier, GSK3B is involved in tau phosphorylation and *β*-amyloid production, two crucial events in the pathogenesis of AD (39). Exposure to electromagnetic fields could alter GSK3B regulation and exacerbate neuronal damage by increasing ROS production and neuroinflammation. This indirect impact on biological processes related to Alzheimer’s underscores the need for further research to clarify how environmental factors, such as radiation exposure, might interact with genetic predisposition to influence the onset of the disease. One of the main concerns is that chronic exposure to 2.4 GHz EMF could induce cumulative damage to the expression of key genes involved in DNA repair and cellular metabolism regulation. This damage could create an environment conducive to synaptic dysfunction and the accumulation of misfolded proteins, characteristic processes of AD (52). Although conclusive evidence of a direct relationship between 2.4 GHz radiation and Alzheimer’s disease is still lacking, the alteration of cellular processes related to oxidative stress and cellular homeostasis presents a plausible avenue for future research. It is important to note that although some of the genes involved in AD, such as APOE and GSK3B, are linked to cellular functions that could be altered by EMF exposure, more research is needed to understand how 2.4 GHz radiation might induce changes in the gene expression of these and other key genes. Figure 8 illustrates that the genes GSK3B and APOE are functionally connected to ITM2B through the APP gene, which plays a crucial role in regulating the processing of the amyloid precursor protein. This protein is a central component in the pathophysiology of Alzheimer’s disease (53, 54). The ITM2B gene interacts directly with APP at the cell membrane and inhibits its cleavage by *β*- and *γ*-secretases, thereby preventing the generation of β-amyloid peptides (Aβ), which are known for their neurotoxicity and their role in the formation of senile plaques characteristic of Alzheimer’s disease (53). In this context, a decrease in ITM2B expression compromises this protective mechanism. Notably, continuous exposure to 2.45 GHz electromagnetic radiation a frequency commonly used in wireless technologies such as Wi-Fi has been reported to significantly reduce ITM2B expression levels in human glial cells (23). This reduction may facilitate the amyloidogenic processing of APP, promoting increased production of Aβ peptides (54). Excessive Aβ leads to its accumulation as extracellular deposits that disrupt synaptic function and contribute to neurodegenerative processes (53, 54). Previous studies have shown that ITM2B deficiency or loss is associated with increased Aβ levels and greater synaptic toxicity, reinforcing its role as a negative regulator of this pathological pathway (53, 54). These findings suggest that ITM2B inhibition induced by exposure to electromagnetic fields may constitute a relevant molecular mechanism linking environmental factors with neurodegenerative diseases (23).

It is worth noting that, although this work addresses the involvement of APP in the neurodegenerative progression induced by electromagnetic fields, it does not delve into the physical mechanism that directly links such exposure to an increase in ion diffusion across the cell membrane. This phenomenon has been addressed in a theoretical study currently underway development (manuscript in preparation), $\begin{array}{l} J_{\text{total}}(z,t)=\{\begin{array}{l} \left[1+m(t)]E_{0}(\frac{k}{\mu_{0}c}+\in_{0}\omega )\cos(\mathrm{kz}-\mathrm{\omega t}\right)\\ -\in_{0}E_{0}\frac{\mathrm{dm}(t)}{\mathrm{dt}}\sin(\mathrm{kz}-\mathrm{\omega t}) \end{array}\}\hat{x}\\ +\sum_{i}P_{i}z_{i}^{2}\frac{V_{m}F^{2}}{\mathrm{RT}}[\frac{{\left(i\right)}_{\text{in}}-{\left(i\right)}_{\text{out}}\cdot \exp(-\frac{z_{i}V_{m}F}{\mathrm{RT}})}{1-\exp(-\frac{z_{i}V_{m}F}{\mathrm{RT}})}]\cdot \hat{n_{i}} \end{array}$ which proposes that electromagnetic induction, under specific conditions of frequency, pulsed modulation, and intensity, can significantly increase the ionic current density in biological media. This phenomenon can be quantitatively described using the Goldman-Hodgkin-Katz (GHK) and Nernst models ($\sum_{i}P_{i}z_{i}^{2}\frac{V_{m}F^{2}}{\mathrm{RT}}[\frac{{\left(i\right)}_{\text{in}}-{\left(i\right)}_{\text{out}}\cdot \exp(-\frac{z_{i}V_{m}F}{\mathrm{RT}})}{1-\exp(-\frac{z_{i}V_{m}F}{\mathrm{RT}})}]\cdot \hat{n_{i}}$), which are fundamental for characterizing ionic flow across cell membranes based on electrochemical gradients and specific ionic permeabilities. This would enhance ionic permeability, disrupt electrochemical equilibrium, and promote sustained membrane depolarization. This dynamic can lead to the opening of voltage-gated ion channels as well as alterations in ion pump function. A representative case is the activation of calcium channels by low frequency pulsed fields, allowing Ca^2+^ influx and the activation of intracellular signaling cascades, facilitating prolonged intracellular accumulation of Ca^2+^ (55, 56). This ion acts as a direct regulator of APP processing by stimulating *β*- and *γ*-secretases, which promote the amyloidogenic pathway and the production of β-amyloid (Aβ) peptides, highly neurotoxic in Alzheimer’s disease (57, 58). Moreover, APP regulates ion channels such as Nav1.6 and KCNQ2/3. A sustained alteration of the ionic gradient may affect its phosphorylation and localization, disrupting its regulatory function and establishing a pathological feedback loop (59). Additionally, excess intracellular Ca^2+^ activates enzymes such as calpains and phospholipases, leading to cellular damage and oxidative stress conditions that further favor the cleavage of APP into toxic products (60). Finally, it has been proposed that Aβ oligomers can form pores in the cell membrane, acting as aberrant ion channels that permit uncontrolled ion influx, particularly of Ca^2+^ (61). This ionic disruption perpetuates the amyloidogenic cleavage of APP, intensifying neurodegeneration. Therefore, an integrated approach that considers both genetic and environmental factors will be essential to assess the potential risk of 2.4 GHz EMF exposure in the development of neurodegenerative diseases such as Alzheimer’s disease.

## Conclusion

In summary, exposure to 2.4 GHz electromagnetic fields emitted by Wi-Fi devices could have an indirect impact on the regulation of genes involved in Alzheimer’s disease, particularly those related to oxidative stress and cellular homeostasis. Although a direct relationship has not been demonstrated, current findings suggest that the alteration of genes such as GSK3B and APOE, which are fundamental in neurodegeneration, could be exacerbated by chronic exposure to this radiation. Future research should address this hypothesis to provide a clearer understanding of the potential risks associated with electromagnetic radiation and its impact on neuronal health and the development of Alzheimer’s disease.

## Data Availability

The data supporting the findings of this study are available in the STRING database and can be accessed at https://string-db.org/.
